# An Examination of the Role of Transcriptional and Posttranscriptional Regulation in Rhabdomyosarcoma

**DOI:** 10.1155/2017/2480375

**Published:** 2017-05-30

**Authors:** Alexander J. Hron, Atsushi Asakura

**Affiliations:** ^1^Stem Cell Institute, University of Minnesota Medical School, Minneapolis, MN 55455, USA; ^2^Paul and Sheila Wellstone Muscular Dystrophy Center, University of Minnesota Medical School, Minneapolis, MN 55455, USA; ^3^Department of Neurology, University of Minnesota Medical School, Minneapolis, MN 55455, USA

## Abstract

Rhabdomyosarcoma (RMS) is an aggressive family of soft tissue tumors that most commonly manifests in children. RMS variants express several skeletal muscle markers, suggesting myogenic stem or progenitor cell origin of RMS. In this review, the roles of both recently identified and well-established microRNAs in RMS are discussed and summarized in a succinct, tabulated format. Additionally, the subtypes of RMS are reviewed along with the involvement of basic helix-loop-helix (bHLH) proteins, Pax proteins, and microRNAs in normal and pathologic myogenesis. Finally, the current and potential future treatment options for RMS are outlined.

## 1. Introduction

Rhabdomyosarcoma (RMS) is an aggressive and malignant form of pediatric cancer developed from myogenic cell lineages, as evidenced by expression of MyoD and desmin. The key to our current understanding of RMS is the role of tissue-specific transcription factors including MyoD, Pax family of proteins, tissue-specific microRNAs (miRNAs), and molecular mechanisms for cell cycle regulation and differentiation governed by these factors.

MyoD is a positively regulating bHLH myogenic regulatory factor (MRF) that acts as a critical control point in conjunction with enhancer box- (E-box-) binding partners and other MRFs including Myf5 and myogenin to commit mesoderm cells to a skeletal muscle lineage [1]. During development and repair, high MyoD expression acts to repress cell renewal, to promote terminal differentiation, and to induce apoptosis [1]. In conjunction with other MRFs, MyoD acts to oppose the role of proliferation-inducing transcription factors including Pax3 and Pax7.

The Pax family of proteins plays an essential role in muscle stem cell maintenance and proliferation. Pax proteins play a nonpeaceful role in fusion protein-positive cases of RMS, where they are thought to contribute in part to its malignant phenotype [2–6]. Together, MyoD and Pax proteins are drivers of the myogenic program and are regulated by multiple factors including miRNAs.

miRNAs are small, noncoding RNAs that are vital to myogenesis and eukaryotic organisms in general due to their ability to posttranscriptionally modify target mRNA [6]. miRNAs function via base pairing with complementary sequences within mRNA molecules. They achieve their silencing effect through a combination of mRNA strand cleavage, reduced translational efficiency in the ribosome, and destabilization of mRNA through poly(A) tail shortening. The effect that the miRNA has on the target mRNA is largely dictated by sequence complementarity, with higher sequence complementarity leading to cleavage of the mRNA and low complementarity leading to reduced translational efficiency [4, 7].

In RMS cells and supportive tissues, key regulatory miRNAs have been disrupted, perhaps partially as a consequence of excessive negative bHLH/E-protein-binding events. Some of these key regulatory miRNAs that have been disrupted include miR-26, miR-27, miR-29, miR-133, miR-181, miR-203, miR-206, miR-214, and miR-378, among others.

Throughout this article, the roles of bHLHs, E-proteins, Pax proteins, and miRNAs in the pathophysiology of RMS are reviewed. Additionally, chromosomal and histological differences between the two major variants are outlined. Finally, current and potential future therapeutic approaches to RMS are explored.

## 2. Rhabdomyosarcoma (RMS)

With nearly 200 new cases being diagnosed yearly in the United States and accounting for 6–8% of all pediatric tumors, RMS is the third most common form of muscle tumor. It is known as a cancer of adolescence due to the majority of new cases being diagnosed in children at or below 14 years of age. More than 50% of new cases occur in children at or below the age of 5, with another, smaller incidence peak in early adolescence [3, 8].

RMS is currently subdivided into embryonal and alveolar variants; each having its own distinct histological, molecular, and genetic markers. Embryonal RMS is the most common form of RMS, with approximately two thirds of all diagnosed RMS cases falling under this category [3]. Embryonal RMS consists of two subtypes, including botryoid RMS and leiomyosarcoma. Histologically, botryoid RMS is denoted by its namesake “grape-like” cell clusters and a dense tumor cell layer under an epithelium (cambium layer) [3]. The leiomyosarcoma form of embryonal RMS often shows up as elongated spindle cells in a storiform pattern [3]. Most embryonal tumors are characterized by their close resemblance to developing skeletal muscle. Additionally, embryonal tumors often display abnormal myoblasts, called rhabdomyoblasts, that have oblong shapes with elliptical nuclei and bland chromatin. Genetically, embryonal RMS is characterized by the loss of heterozygosity at the 11p15 locus, a region of chromosome 11 harboring the insulin-like growth factor 2 (IGF2) gene and is associated with the loss of maternal and copying of paternal chromosomal materials [3]. Alveolar RMS tissue is characterized by the appearance of small, round, densely packed cells that are arranged in such a manner that they resemble pulmonary alveoli, with an empty space in the center of the cluster. There is also a solid variant, which belongs to the alveolar variant, but does not have the characteristic empty space in the middle of the cluster [3]. The solid variant of alveolar RMS can make it difficult to tell the difference between embryonal and alveolar RMS through histology alone. However, alveolar RMS cells often tend to be larger, with centrally located nuclei and less cytoplasm than cells of the embryonal RMS variant [3]. Prognostically, embryonal RMS variants are associated with a limited stage disease and a favorable outcome. On the other hand, alveolar RMS variants are linked with a less favorable prognosis [9]. Currently, few effective, targeted treatment options exist for RMS; however, research is being done to determine potential future treatment options.

## 3. Chromosomal Translocations and Fusion Proteins in RMS

In terms of molecular and genetic markers of embryonal and alveolar rhabdomyosarcoma, 80–90% of alveolar RMS cases have chromosomal translocations of the DNA-binding domain of PAX3 or PAX7 at 2q35 to the transactivation domain of the FOXO1 gene at t(2;13) (q35;q14) or t(1;13) (p36;q14), respectively [3, 10–14]. This typically results in the formation of a fusion protein between PAX3 or PAX7 and FOXO1 in alveolar RMS, although PAX7-FOXO1 fusion is much less common and less potent than the PAX3-FOXO1 fusion protein form [3, 14]. Both members of the paired box type homeobox transcription factor family, Pax3 and Pax7, are involved in neurogenesis, cardiogenesis, melanoma cell pathophysiology, and myogenesis during development. Pax3 gene mutant mice have shown the essential roles of Pax3 in several developmental systems including embryonic myogenesis and muscle satellite cell differentiation by regulating gene expression of cMET. cMET is a hepatocyte growth factor/scatter factor (HGF/SF) receptor required for myogenic progenitor cell migration, with Bcl-2 and Bcl-xl serving antiapoptotic functions [13, 15, 16]. In contrast, Pax7 is required for specification of muscle satellite cells and myogenic stem cells and essential for postnatal muscle growth and regeneration [17, 18]. FOXO1 is a member of the forkhead/HNF-3 transcription factor family. The chimeric protein of PAX3-FOXO1 is a more potent transcriptional activator than wild-type Pax3. Ectopic expression of the chimeric gene converts fibroblasts to myogenic cells by the activation of multiple muscle-specific genes [19, 20]. These observations indicate that the overexpression of growth factors such as IGF2 or the activation of Pax genes may result in RMS.

## 4. bHLH/E-Protein Heterodimers in RMS

An increasingly relevant family of proteins to developmental biology, the bHLH family of transcription factors, has gained considerable attention, especially in myogenesis-related research. bHLH proteins including MyoD, Myf5, and musculin (MSC)/MyoR are vital for the regulation of the differentiation program that takes place in skeletal muscle cells [21]. They act through direct binding to promoters upstream of target gene sequences, as well as through heterodimer formation with E-proteins [22]. Depending on the characteristics of the bHLH protein that eventually binds the E-box through either of these mechanisms, myogenesis can either be initiated or inhibited [23]. Based on the effects of the bHLH protein, it can be classified as a negative bHLH or a positive bHLH. Positive bHLHs, such as MyoD and Myf5, upregulate target sequences, whereas negative bHLHs, such as MSC, downregulate them. Contrary to the roles of proliferation-inducing transcription factors such as PAX3 and PAX7, MyoD acts to end the proliferative phase and begin the differentiation into skeletal muscle. One study of interest by Tapscott et al. found that in a genome-wide binding comparison of MyoD in normal human myogenic cells versus RMS cells, MyoD bound to the same areas in both cell types. However, MyoD exhibited poor binding at a subset of myogenic genes often underexpressed in RMS cells, including RUNX1, MEF2C, JDP2, and NFIC. Further, when these genes were re-expressed, myogenesis was rescued [24].

In normal tissue, MyoD can bind either directly to E-boxes upstream of target sequences or through dimerization with a full-length E-protein to these same sites. The ultimate binding of MyoD:E-protein heterodimers to the E-box in normal tissue is also regulated through competitive inhibition with negative bHLHs that are present in varying amounts during different stages of differentiation. In normal tissue, the level of competition between MyoD and negative bHLHs for E-proteins is relatively low compared to the RMS model [25]. Current research suggests that negative bHLHs, such as MSC, found in RMS tissue compete with MyoD and other positive bHLHs to a much greater extent for binding with E-proteins. Subsequently, affected cells remain in a stage between muscle precursors and terminally differentiated skeletal muscle. Proteins that are competing for binding with full-length E-proteins include negative bHLHs, such as MSC, and the splice form of the full-length E-protein, E2A-2/5. These two influences act synergistically through different mechanisms to ultimately decrease the transcription of genes that are key to the process of myogenic differentiation. Negative bHLH transcription factors, such as the myogenic inhibitory factor MSC, compete with MyoD for dimerization with full-length E2A proteins. When MSC:E2A heterodimers form, they bind to the E-box upstream of target sequences and downregulate downstream regions including MyoD gene. This maintains a tissue form intermediate between proliferating muscle precursors and fully differentiated skeletal muscle. Additionally, MSC likely plays an opposing role to MyoD, as it shows substantial overlap in binding when analyzed through genome-wide studies [26].

The splice form of the E2A protein, the E2A-2/5 splice variant, also competes with positive and negative bHLHs alike for binding with the full-length E2A protein. In recent in vitro studies, gel shift assays were used to determine the binding potential of the E2A-2/5 splice form and the full-length E2A protein. Based on the results of the study, E2A-2/5 splice forms have the potential to bind full-length E2A proteins in in vitro gel shift assays [25]. In vivo, it is thought that the E2A-2/5 splice variant competes with positive and negative bHLHs for binding with the full-length E2A protein. The resulting E2A:E2A-2/5 heterodimers likely do not bind the e-box; instead, the E2A-2/5 protein acts to sequester full-length E2A proteins that are present in the cell so that the upregulation of target regions is unable to occur because of the diminished amounts of E2A:MyoD heterodimers. Current research has uncovered that MyoD:E2A heterodimer levels are lower and are antagonized by negative bHLHs and E2A-2/5 splice forms to a greater extent than in normal tissues [25]. Taken together with Pax fusion proteins and microRNA dysregulation, this molecular mechanism likely contributes to the pathophysiology of RMS.

## 5. Posttranscriptional Control in RMS through Muscle-Specific miRNAs (myomiRs) (Table 1)

Beginning with the discovery of the first canonical miRNA in *C. elegans*, lin-4, miRNA function in eukaryotes has become an increasingly important and relevant topic for researchers [27–29]. Within the RMS disease field, miRNAs have gained new attention not only as important contributors to the disease but also as potential therapeutic targets. miRNAs have no protein product and are encoded by specific sequences downstream of promoters. When activated, the miRNA sequence is transcribed then processed initially in the nucleus by the RNase III enzyme Drosha, which removes the 5′ cap and poly(A) tail [30, 31]. Afterwards, the pre-miRNA is passed out of the nucleus into the cytoplasm, where further processing by dicer enzymes converts the pre-miRNA into the final miRNA molecule [32, 33]. This molecule then incorporates into an RNA-induced silencing complex (RISC) with another protein which aids in binding to target mRNAs [34]. Depending on sequence consensus between the miRNA and the target region of the mRNA, the mRNA will either be degraded (high consensus) or translationally inhibited due to the RISC present on the mRNA (low consensus) [7]. This mechanism is especially important because it provides ways in which the cell can control protein production posttranscriptionally, which allows multilayered regulation of gene expression. Depending on tissue type, various miRNAs are more abundant than others. In the case of skeletal muscle-specific miRNAs (myomiRs), miR-1, miR-206, and miR-133a are common, with each playing regulatory roles integral to myogenesis. Myogenic dysfunction in RMS tissues is exacerbated by deregulated miRNA levels, which have in many cases been found to be lower than in adjacent skeletal muscle tissue. At low levels, miRNAs have less of a repressive effect on their target genes, opening tissue up to potential problems including cancer.

Perhaps the most studied myomiR is miR-206. MiR-206 is currently known to target cMet, which is a proto-oncogene receptor overexpressed in a variety of cancers, including RMS. cMet levels in RMS tissue have been found to be inversely related to miR-1/206 levels, and various studies utilizing this knowledge have shown that MET is a key target for the anticancer effects of miR-1/miR-206 [35]. This leads to the possibility that restoration of miR-1/miR-206 to normal physiological levels may provide therapeutic potential for RMS. Indeed, this potential has been tested in mice with xenografted, lentivirus-infected RD cells, an RMS cell line, expressing either miR-1, miR-206, or the negative control. Transient transfection of miR-1/206 into cultured RD cells led to a significant decrease in cell growth and migration. Additional findings from this study revealed that the differences in tumor volume were apparent between miR-1/206-expressing tumor cells and the negative control, with miR-1/206-expressing tumor cells displaying growth delay in comparison with the negative control [35].

miRNAs that are predominantly expressed in other tissue types also play a role in RMS. Among these, miR-26, miR-27, miR-29, and miR-181 play roles in myogenesis and have all been shown to be deregulated in RMS [36]. miR-26a has been shown to have a positive effect on myogenesis by targeting the histone methyltransferase enhancer of zeste homolog 2 (Ezh2) [37, 38]. Ezh2 is an enzyme in humans that aids in maintaining closed chromatin structures that prevent the transcription of key developmental genes. It performs this role through the trimethylation of lysine 27 of histone 3, resulting in chromatin condensation and thus transcriptional repression of target genes. Acting through this mechanism, Ezh2 inhibits myogenesis by repressing late-stage muscle-specific genes such as muscle creatine kinase (MCK) and myosin heavy chain (MHC) [39, 40].

Another crucial myomiR that is currently undergoing scientific studies is miR-29, which is regulated by NF-*κ*B acting through YY1 and the polycomb group. In many muscle tumors, including RMS, miR-29 has been shown to be downregulated in part due to an elevation in NF-*κ*B and YY1, leading to a decrease in likelihood that the cell will undergo differentiation [36]. Wang et al. also showed that in immunocompromised mice with RH30 tumors, injection of miR-29b-expressing virus intratumorally resulted in tumors that displayed slower growth. Between eight days postinjection and the experimental end point, the average size of the control tumor was 1.9 times larger than the miR-29b tumor [41].

Another important group of miRNAs in RMS pathology is the miR-181a/miR-181b gene cluster. During normal myogenesis, the homeobox gene HoxA11 initially inhibits myogenesis. In order for myogenesis to occur, this gene must be downregulated. The miR-181a/miR-181b gene cluster is able to do just that by inhibiting the expression of HoxA11, which allows for terminal differentiation to occur. In most cases of RMS, miR-181 is downregulated and is unable to exert a repressive role on HoxA11, which effectively prevents RMS cells from differentiating [42].

As more is learned about the various miRNAs that contribute to the RMS phenotype, epigenetic miRNA control mechanisms are being examined. One such miRNA in which epigenetic controls are at work is miR-203. miR-203 directly targets p63 and leukemia inhibitory factor (LIF) in RMS cells. Targeting of these factors then promotes myogenic differentiation via the inhibition of the Notch and JAK/STAT pathway, respectively. In both RMS biopsies and various RMS cell lines, miR-203 was found to be downregulated due to promoter hypermethylation. Interestingly, miR-203 function was found to be restored after exposure to DNA-demethylation agents. Further, this led to a reduction in migration and proliferation as well as the promotion of terminal myogenic differentiation [43].

miR-214 has also been shown to be downregulated in human RMS cell lines. miR-214 exerts its suppressive role in mouse embryonic fibroblasts (MEFs) by suppressing their proliferation. After the introduction to RD cells, it was shown to have a repressive effect on tumor cell growth and culture colony formation and a stimulatory effect on myogenic differentiation, apoptosis, and xenograft tumorigenesis. miR-214 was shown to exert its inhibitory effects on the proto-oncogene N-ras. In MEF *miR-214^−/−^* cells, N-ras was found to be elevated. Additionally, in control cells, forced expression of N-ras from cDNA lacking a 3′-untranslated region neutralized the antiproliferative and promyogenic activities of miR-214 [44].

One final miRNA of interest is miR-378. Like many of the miRNAs described thus far, it has been found to be downregulated in RMS cells. In one study by Megiorni et al., the expression level of 685 miRNAs was investigated via a deep-sequencing approach, where miRNA expression across various RMS cell lines was investigated. In their study, they found that miR-387 was, on average, downregulated and that it may function as a tumor suppressor in RMS. Further, they posited that restoration of miR-387 expression could provide therapeutic benefits [45].

## 6. miRNA-Mediated Pax3 Regulation in RMS and Muscle Stem Cell Maintenance

Pax3 expression is subject to posttranscriptional regulation, and timely downregulation of Pax3 expression is crucial for myogenic differentiation. Recent work demonstrates that Pax3 expression is regulated by multiple stages, including ubiquitination-mediated protein degradation, Staufen 1-mediated mRNA decay, and miR-27b-mediated translational inhibition [46–48]. During embryonic myogenesis, both types of miR-27 (miR-27a and miR-27b) target the 3′UTR of PAX3, an important transcription factor for myoblast proliferation, in order to downregulate PAX3 expression. This leads to a shift from PAX3-positive cells to myogenin-positive cells, indicating a transition from a predominantly proliferative state to differentiation. We have recently demonstrated that MyoD negatively regulates Pax3 gene expression through the action of miRNAs. Because Pax3 functions as a cell fate determination factor and for maintenance of the undifferentiated state in muscle and melanocyte stem cells, downregulation of Pax3 is essential for terminal differentiation, which is also accompanied by apoptosis. We also noticed that Pax3 is a survival factor that transcriptionally activates the antiapoptotic genes Bcl-2 and Bcl-xL [16]. Therefore, negative regulation of Pax3 expression by MyoD-regulated miRNAs is a critical point for MyoD-dependent apoptosis in myoblasts. Experiments from gene knockout mice demonstrate that Pax3 functions as a survival factor during embryogenesis [49–51]. It has been reported that Pax3 positively regulates Bcl-xL gene expression by binding to the 5′-flanking region of the Bcl-xL gene [52]. Previously, screening of binding proteins for the 1 kb Bcl-2 promoter identified 43 different transcription factors including Pax3 [53]. We demonstrate that Pax3 positively regulates Bcl-2 gene expression via the 5′-flanking region of this gene, strongly indicating that Pax3 functions as an antiapoptotic factor by transcriptionally upregulating Bcl-2 and Bcl-xL gene expression. Pax3 also facilitates the malignant progression of RMS and melanomas [54–56]. Overexpression of MyoD or inhibition of Pax3 by miRNAs may induce apoptosis in RMS and neuroblastoma cells, which may provide a novel anticancer therapy for associated tumors [2, 5, 57, 58].

**Figure 1:**
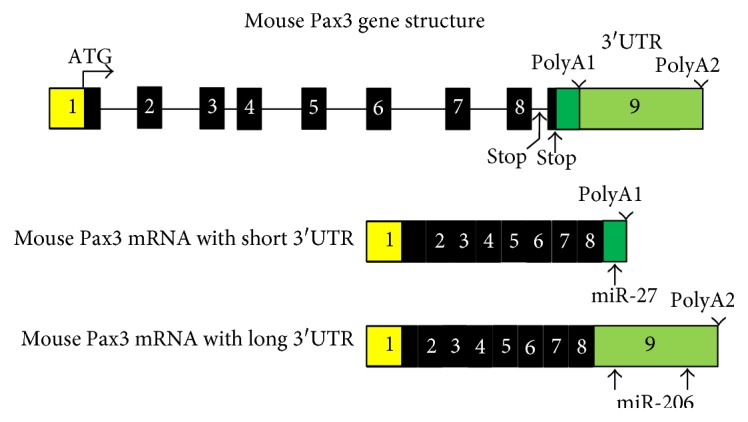
Pax3 3′UTR contains microRNA-binding sites. Mouse Pax3 gene and mRNA structures. Numbered boxes denote each exon. White boxes denote the 5′UTR and the shorter 3′UTR. Black boxes denote coding regions. There are 2 stop codons and 2 polyA signal sequences (polyA1 and polyA2) in mouse Pax3 gene, leading alternative polyadenylation. The right side white box denotes the shorter 3′UTR containing miR-27-binding site. The gray box denotes the longer 3′UTR containing two miR-1-/miR-206-binding sites.

Adult skeletal muscle possesses extraordinary regeneration capabilities. After exercise or muscle injury, large numbers of new muscle fibers are normally formed within a week because of expansion and differentiation of muscle satellite cells [59]. Satellite cells are a small population of myogenic stem cells for muscle regeneration which are normally mitotically quiescent. Following injury, satellite cells initiate proliferation to produce myogenic precursor cells, or myoblasts, to mediate the regeneration of muscle [60–62]. The myoblasts undergo multiple rounds of cell division prior to terminal differentiation and formation of multinucleated myotubes by cell fusion. Pax3 together with expression of Pax7 and downregulation of MyoD is detected in a subset of satellite cells and potentially important for muscle stem cell maintenance and self-renewal [46, 63–66]. For mouse Pax3, there are two putative polyA signal sequences in the 3′UTR. Both proximal (polyA1) and distal (polyA2) polyA signal sequences were indeed used for transcription of Pax3 mRNAs with the shorter and longer 3′UTRs, respectively (Figure 1). The shorter 3′UTR contains a miR-27-binding site, and the longer 3′UTR contains both putative miR-1- and miR-206-binding sites [16, 48, 67]. In contrast, the human Pax3 gene only contains the polyA2 sequence, and thus, the human Pax3 mRNA contains the longer 3′UTR with the two putative miR-1-/miR-206-binding sites [68, 69]. Recent work showed that quiescent satellite cells (QSCs) express high levels of Pax3 and miR-206 [67]. In these QSCs, Pax3 transcripts possess shorter 3′UTRs that render them resistant to suppression by miR-206, which is important in maintaining muscle stem cell status in skeletal muscle. These results suggest alternative polyA signals in circumventing miRNA-mediated regulation of muscle stem cell function including stem cell self-renewal and maintenance.

**Figure 2:**
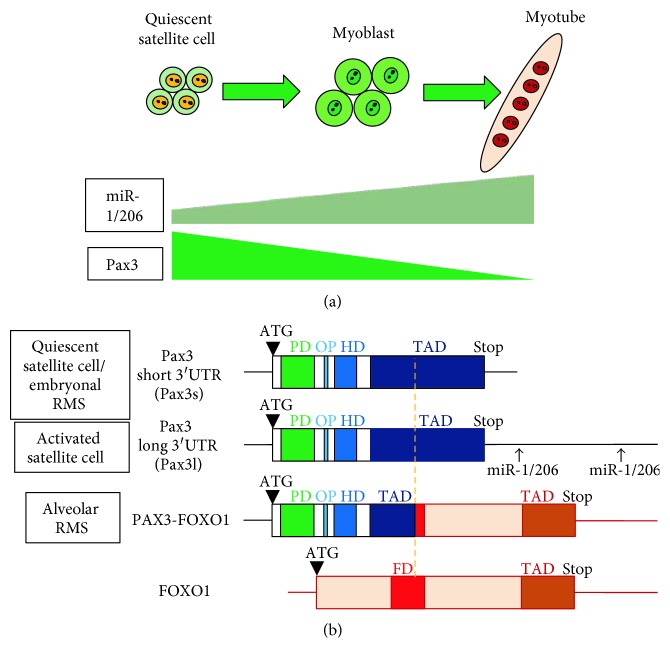
Truncation and loss of Pax3 3′UTR during muscle stem cell self-renewal and RMS progression. (a) Schematic model of Pax3 and miR-1/206 expression during muscle stem cell self-renewal and activation. (b) Mouse Pax3 mRNA structures with short and long 3′UTRs and human RMS-derived PAX3-FOXO1 fusion gene.

Both miR-1 and miR-206 expressions are downregulated in RMS compared to normal skeletal muscle but still much higher than nonmuscle tissues, supporting the myogenic origin of RMS. In alveolar RMS, the chromosomal translocation-generated PAX3-FOXO1 fusion protein is a superactive transcription factor due to the activation domain of FOXO1 and thus promotes RMS proliferation and progression. In addition, PAX3-FOXO1 fusion gene lost Pax3-3′UTR due to the translocation as shown in Figure 2. Therefore, PAX3-FOXO1 fusion gene is no longer the target of miR-1/206, which may lead to an increased expression level of this fusion gene. In embryonal RMS, Pax3 is not associated with chromosomal translocation, but there are Pax3 3′UTR abnormalities including shorter transcript variants lacking miR-1/206-binding sites [70], escaping the miR-1/206-mediated Pax3 gene suppression as seen in the QSCs (Figure 2). Therefore, there are common molecular mechanisms in Pax3 gene regulation in both muscle stem cell self-renewal and RMS progression.

## 7. Therapies and Approaches

Like many cancers, RMS can carry a dismal prognosis, especially in cases where the alveolar variant is displayed. Treatment options that currently exist include surgical removal of affected tissues, chemotherapy, radiation, or these treatments in combination [71, 72]. In some cases of RMS, surgical excision may be recommended. This is an effective treatment option in cases where the cancer has not metastasized to other tissues. Often, large portions of affected tissue can be resected; however, microscopic margins may remain. Tumor resection, followed by a combination of intensive chemotherapy and radiation, can help to suppress and kill unresected portions [73, 74]. Although current treatment options are effective in some cases, they continue to be a nonideal treatment option for patients with RMS. With ongoing research into the molecular mechanisms at place in RMS, more advanced and effective treatment options for RMS may begin to emerge.

By researching the roles of bHLH transcription factors in myogenesis along with the regulatory roles of miRNAs, more effective treatment methods for RMS can be elucidated. Common to all forms of RMS is that the tissue is in an intermediate state between muscle precursor cells and terminally differentiated muscle. This leaves determining a potential treatment option square in the lap of developmental biologists and stem cell researchers, specifically those studying diseases of skeletal muscle. One common idea among many stem cell researchers is that it may be possible to coax the RMS tissues to differentiate into muscle fibers, thus losing their tumorigenic and metastatic potential [73, 74].

One promising method for coaxing differentiation of these muscle precursor-like cells is to use RNA interference methods, such as miRNAs and siRNAs, to force differentiation to occur. This could be put into practice by introducing a miRNA or siRNA that posttranscriptionally modulates MSC mRNA so that it does not have the chance to compete with MyoD and Myf5 for E-protein dimerization, which might ultimately lead to increased transcription of MyoD target genes, thus inducing myogenic differentiation with subsequent loss of proliferative capacity. An important area that needs further research before RNA interference methods could be used on human patients would to be to determine what genes a certain miRNA represses in addition to the target gene, as most miRNAs lack the specificity of siRNAs. Another option for inducing terminal differentiation would be to use gene therapy to insert another gene for the MyoD protein into RMS patients. This would theoretically cause a twofold increase in the amount of MyoD that is present in the cell, leading to increased competition with negative bHLHs such as MSC. This would also lead to increased competition with inhibitory E2A splice forms such as E2A-2/5. Yet another treatment option might involve using protein therapies to induce differentiation. Proteins could be used for treatment of RMS in multiple ways, either as negative bHLH-binding proteins or as supplements to the existing positive bHLHs that are present in the cell. One example of how this therapy could be used would be to introduce a protein into the RMS patient that binds to MSC and/or other negative bHLHs in RMS tissues and renders them inactive and unable to bind to full-length E2A proteins, allowing for MyoD to have a more profound effect in these tissues.

miR-206, as described earlier, has been shown to inhibit human rhabdomyosarcoma growth in xenotransplanted mice by promoting tumor differentiation [75]. Similarly, miR-29b, also described earlier in this article, was shown to slow tumor growth in immunocompromised mice with RH30 tumors. Between an eight-day postinjection and the experimental end point, the average size of the control tumor was 1.9 times larger than the miR-29b tumor [41]. Based on results of these studies and others in this article, translation of these therapies into clinical trials may have some merit after safety evaluation and delivery verification.

As seen throughout this review, experiments in xenotransplanted mice with microRNAs have shown slowed tumor growth and increased differentiation of cells from an arrested myoblast phase state. The combination of current and past research in this field has led to a climate in which discovering new treatments may be just around the corner. However, even as the scientific community continues to discover new molecular targets, it is important to keep in mind that further challenges still exist in finding therapeutic options, including identifying reliable and reproducible delivery methods and evaluating safety and efficacy in human patients.

## Figures and Tables

**Table 1:** Deregulated miRNAs, their roles, targets, and expression in both alveolar and embryonic RMS.

Name	miRNA level in RMS relative to normal human myoblasts		Target genes in RMS	Function	Reference
	Alveolar	Embryonal			
miR-1	Down	Up	CCND2, cMET, PAX3	Tumor suppressor	[35, 36]
miR-24	Down	Down	—	—	[76]
miR-26a	Down	Down	Ezh2	Tumor suppressor	[36, 37, 76]
miR-27a	Down	Down	PAX3	Tumor suppressor	[36, 76]
miR-29	Down	Down	CCND2, PAX3, CCND2	Tumor suppressor	[36, 41]
miR-133a	Down	Down	TPM4	Tumor suppressor	[36, 76, 77]
miR-133b	Down	Down	—	Tumor suppressor	[36, 75]
miR-181	Down	Down	HOXA11	Tumor suppressor	[36, 42]
miR-183	Up	—	EGR1, PTEN	Oncogene	[36, 77]
miR-203	Down	Down	p63, LIF	Tumor suppressor	[43, 76]
miR-206	Down	Down	CCND2, cMET, PAX3	Tumor suppressor	[35, 75, 77]
miR-214	Down	Down	N-RAS	Tumor suppressor	[38, 44]
miR-301	Up	Up	—	Oncogene	[76]
miR-378a	Down	Down	IGF1R	Tumor suppressor	[45]
miR-450b	Down	Down	ENOX2, PAX9	Tumor suppressor	[44, 78]
miR-485	Up	—	NF-YB	Oncogene	[79]

## Abbreviations

bHLH: Basic helix-loop-helix
E-protein: E-box-binding protein
EZH2: Enhancer of zeste homolog 2
FOXO1: Forkhead box protein O1
HoxA11: Homeobox A11
miR: MicroRNA
miRNA: MicroRNA
MSC: Musculin
myomiR: Myofiber microRNA
PAX: Paired box
RMS: Rhabdomyosarcoma
siRNA: Short interfering RNA.
